# Recruiting and Screening Older Adults With Alzheimer’s Disease for the FIT-AD Trial

**DOI:** 10.1093/geroni/igab046.1771

**Published:** 2021-12-17

**Authors:** Fang Yu, Jean Wyman, Susan Greimel, Lin Zhang

**Affiliations:** 1 Arizona State University, Phoenix, Arizona, United States; 2 University of Minnesota, Minneapolis, Minnesota, United States; 3 University of Minnesota, University of Minnesota, Minnesota, United States

## Abstract

Recruiting older adults with Alzheimer’s disease (AD) into clinical trials has been very challenging even for resource-rich trials. This presentation will discuss the recruitment rate, screening ratio, and recruitment yield and costs in the FIT-AD Trial. The FIT-AD Trial was a single-site, pilot randomized controlled trial testing the effects of 6-month aerobic exercise on cognition and hippocampal volume in community-dwelling older adults with mild-to-moderate AD dementia. Ten recruitment strategies and a 4-step screening process were used to ensure a homogenous sample and exercise safety. The target sample size was 90. During the 48-month recruitment period, 396 individuals responded to our recruitment, 301 were reached, and 103 were tentatively qualified at step 4. Of these 103, 67 (69.8%) completed the optional magnetic resonance imaging (MRI) component of the trial and 7 were excluded due to abnormal MRIs. In year 4, our sample size was increased to allow individuals in the screening process a chance to enroll, resulting in a final sample size of 96. Per enrolled participant, the recruitment rate was 2.15, the screen ratio was 2.92, and the recruitment yield was 31.9%. Over 49% of the enrolled participants were yielded through referrals (28.1%) and Alzheimer’s Association events/services (21.9%). The total recruitment cost was $38,246 ($398 per randomized participant). The results indicate that a multi-prong, extensive community outreach-based approach is essential in recruiting older adults with AD dementia into an exercise trial. Referral was the most cost-effective strategy. Two individuals needed to be screened to enroll one participant.

